# Adult primary paratesticular mesenchymal tumors with emphasis on a case presentation and discussion of spermatic cord leiomyosarcoma

**DOI:** 10.1186/1746-1596-9-90

**Published:** 2014-05-06

**Authors:** Andrea B Galosi, Marina Scarpelli, Roberta Mazzucchelli, Antonio Lopez-Beltran, Lucio Giustini, Liang Cheng, Rodolfo Montironi

**Affiliations:** 1 Urology Division, “Augusto Murri” General Hospital, Fermo, Italy; 2 Section of Pathological Anatomy, Polytechnic University of the Marche Region, School of Medicine, United Hospitals, Via Conca 71, I - 60126 Torrette, Ancona, Italy; 3 Unit of Anatomic Pathology, Department of Surgery, Faculty of Medicine, Cordoba, Spain; 4 Fundação Champalimaud, Lisbon, Portugal; 5 Oncology Division, “Augusto Murri” General Hospital, Fermo, Italy; 6 Department of Pathology and Laboratory Medicine, Indiana University School of Medicine, Indianapolis, IN, USA

**Keywords:** Leiomyosarcoma of the spermatic cord, Mesenchymal tumors of the scrotum, Spermatic cord, Liposarcoma, Malignant fibrous histiocytomas, Rhabdomyosarcoma

## Abstract

**Background:**

The aim of this report is related to adult primary paratesticular mesenchymal tumors with emphasis on a case presentation and discussion of the spermatic cord leiomyosarcoma. Primary paratesticular tumors are rare, only accounting for 7% to 10% of all intrascrotal tumors. In adults, more than 75% of these lesions arise from the spermatic cord, 20% being leiomyosarcoma. Tumor grade, stage, histologic type, and lymph node involvement are independently predictive of prognosis.

**Findings:**

The case report concerns a 81-year-old man presented with a 3-year history of painless lump in the right hemiscrotum. Scrotal examination demonstrated a 5.1-cm, firm-to-hard mass attached to the spermatic cord. Scrotal ultrasound scan revealed a heterogeneous mass separate from the testis. He was treated with an radical orchi-funicolectomy. Histologically the lesion is composed of spindled cells with often elongated, blunt-ended nuclei and variably eosinophilic cytoplasm. Areas with pleomorphic morphology are present. The level of mitotic activity is equal to 3/10 HPF in the areas with spindle cell morphology and to 12/10 HPF in the areas with pleomorphic morphology. The final diagnosis was that a leiomyosarcoma of the spermatic cord, with grade 1 and grade 2 areas, stage pT2b cN0 and cM0. The patient has been followed up for 3 months with CT scans and shows no signs of recurrence.

**Conclusions:**

Spermatic cord leiomyosarcoma, although rare, should be one of the first differential diagnoses for a firm-to-hard lump in the cord. Apart from radical orchi-funicolectomy, there has been added benefit of adjuvant radiotherapy to prevent any loco-regional lymph node recurrence.

**Virtual Slides:**

The virtual slide(s) for this article can be found here: http://www.diagnosticpathology.diagnomx.eu/vs/1613030331125632

## Background

### Primary paratesticular mesenchymal tumors

Primary paratesticular tumors are rare, only accounting for 7% to 10% of all intrascrotal tumors. In adults, more than 75% of these lesions arise from the spermatic cord [1-3]. The most frequently reported benign tumors are hemangiomas, lymphangiomas, leiomyomas and lipomas. Male angiomyofibroblastoma-like tumor is a benign tumor occurring in the scrotum or inguinal region of older men. Other benign lesions include a variety of nerve sheath tumors.

Among the malignant tumors, the most common histotype is liposarcoma (46.4%), followed by leiomyosarcoma (LMS) (20%), malignant fibrous histiocytomas (MFH) (13%), and embryonal rhabdomyosarcoma (9%) [4]. Liposarcomas and MFH have similar age distribution; some tumors historically diagnosed as the latter actually represent dedifferentiated liposarcomas. Liposarcoma and MFH occur predominantly in older men, 75% of these tumors being reported between the ages of 50–80 years. Concerning LMS, the peak incidence is in the sixth and seventh decade. Embryonal rhabdomyosarcoma is the most common malignant mesenchymal tumor in children [5,6].

#### Clinical features

Most scrotal tumors are deep-seated. Benign lesions may present as slowly enlarging, asymptomatic or mildly uncomfortable masses. Benign lesions may present as slowly enlarging, asymptomatic or mildly uncomfortable masses. In general, malignant tumors are more likely to be symptomatic, large, and have a history of rapid growth. Liposarcoma is most common, while LMS and MFH histologic subtypes are observed to be the most aggressive [4]. Tumor grade, stage, histologic type, and lymph node involvement are predictive of prognosis [4,7-9]. Low-grade LMSs have a good prognosis, whereas high grade tumors often develop metastases and have a significant tumor-related mortality. Paratesticular liposarcoma tends to have a protracted course with common recurrences. Dedifferentiation occurs in a minority of cases and also tend to have a protracted clinical course with local recurrences, although distant metastases may also occur [10].

#### Imaging

Liposarcomas generally present as large extratesticular masses, which are often solid and hyperechoic by ultrasound. However, the sonographic appearance of these tumors is variable and non-specific. CT and MR imaging are much more specific with fat being easily recognized with both modalities [11,12]. By CT, fat will appear very low density similar to subcutaneous fat. On MR imaging the fat in a liposarcoma will follow the signal intensity of surrounding fat on all imaging sequences. Additionally a fat-suppressed imaging sequence should be performed for confirmation. Fat will lose signal intensity on this sequence. Lipomas and hernias containing omentum are potential mimics, but the former are generally smaller and more homogeneous, and the latter are elongated masses, which can be traced back to the inguinal canal.

With the exception of liposarcoma, none of the other sarcomas can be differentiated from one another radiologically. They all tend to be large, complex, solid masses [11]. Because of their large size, their extent is better demonstrated by CT and MR imaging rather than ultrasound. However, ultrasound is the first exam in the clinical management of all scrotal masses and it has a key role in the initial management.

#### Histopathology

Hemangiomas are most common within the scrotum. Granular cell tumours of the scrotum may be multifocal and are similar to those elsewhere in the skin. Calcifying fibrous (pseudo)tumor is a densely collagenous, paucicellullar fibroblastic lesion with focal lymphoplasmacytic infiltration and scattered psammomatous calcifications.

Great majority of liposarcomas are well-differentiated with various combinations of lipoma-like and sclerosing patterns. Multivacuolated lipoblasts may be present, but are not required for diagnosis. Presence of significant nuclear atypia in adipocytes is decisive. Dedifferentiation to spindle cell “fibrosarcoma-like” or pleomorphic “MFH-like” phenotype occurs in a proportion of paratesticular liposarcomas [10]. This component can give rise to metastases. MFH, a pleomorphic fibroblastic-myofibroblastic sarcoma, is usually diagnosed by exclusion.

Leiomyomas are composed of mature smooth muscle cells. Focal nuclear atypia may occur, but the presence of prominent atypia should lead to a careful search for mitotic activity or coagulation necrosis which are features of LMS. LMSs are typically composed of spindled cells with often elongated, blunt-ended nuclei and variably eosinophilic cytoplasm. Areas with round cell or pleomorphic morphology may occur. The level of mitotic activity varies widely, from <1 mitotic figures per 10 high power fields (HPF) to >70 mitotic figures per 10 HPF [13].

## Findigs

**Figure 1:**
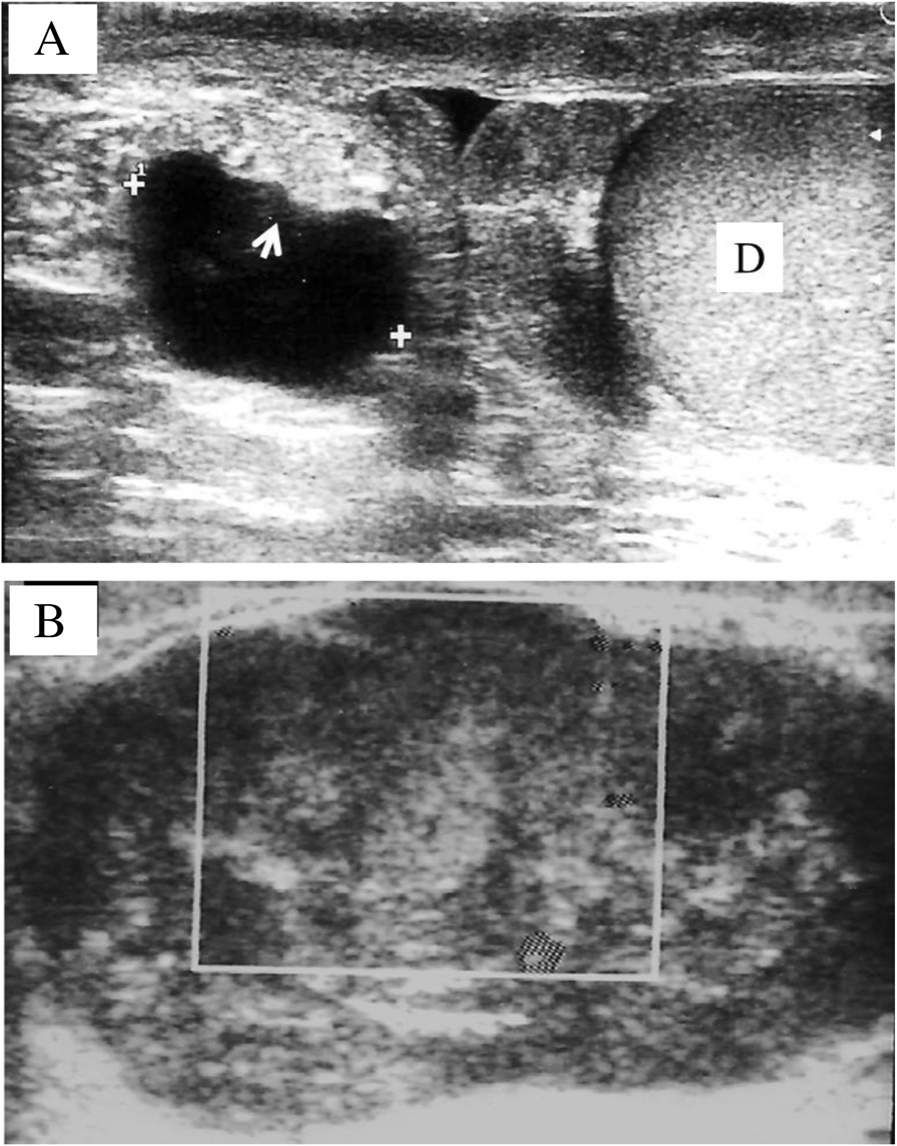
**Scrotal ultrasound. A**. 2-cm lesion with cystic appearance and intracystic material (Arrow), close to the head of the epidydimis, and separated from the didimis (D). **B**. Preoperative scrotal untrasound shows a heterogeneous mass.

A 81-year-old man presented with a 3-year history of painless lump in his right hemiscrotum. A scrotal ultrasound examination performed approximately two years ago showed a 2-cm lesion with cystic appearance (Figure 1A) with intracystic material, close to the head of the epidydimis, and separated from the didimis. A very recent scrotal examination demonstrated a 5-cm firm-to-hard mass. The mass had regular surface without any attachment to dartos and surrounding tissues. Ultrasound scan revealed a heterogeneous mass separate from the didimis (Figure 1B), close to the epidydimis head, attached to the spermatic cord. Echo-power-doppler ultrasound reveals solid tissue with vascular signals inside the lesion. The patient underwent radical orchi-funicolectomy following a sub-inguinal approach. The postoperative course was uneventful. The procedure for this research project conforms to the provisions of the Declaration of Helsinki. Consent was obtained from the patient’s next of kin for the publication of this report.

**Figure 2:**
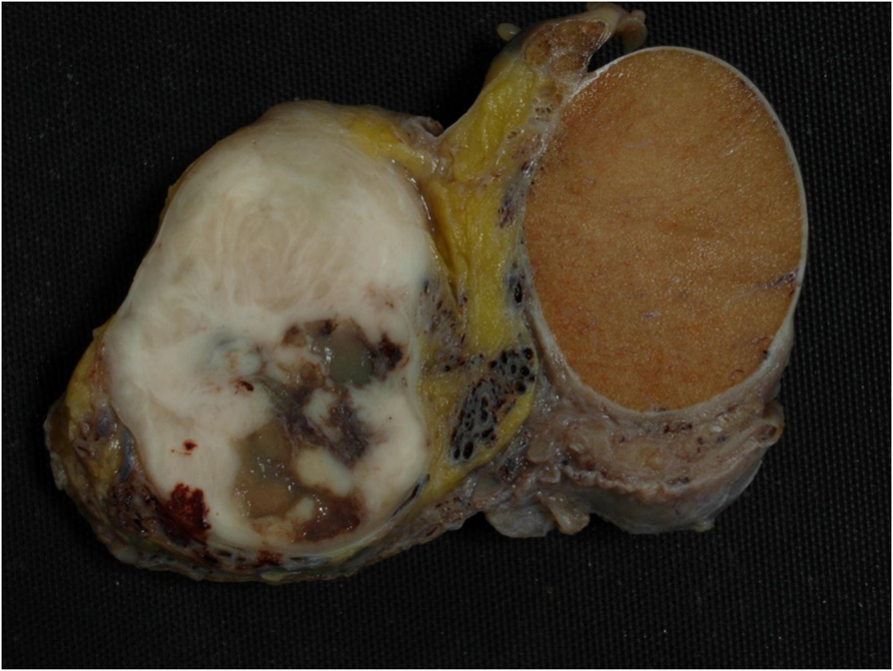
Gross appearance of the neoplasm. On the cut surface the lesion is whitish in color with mucoid-like areas.

**Figure 3:**
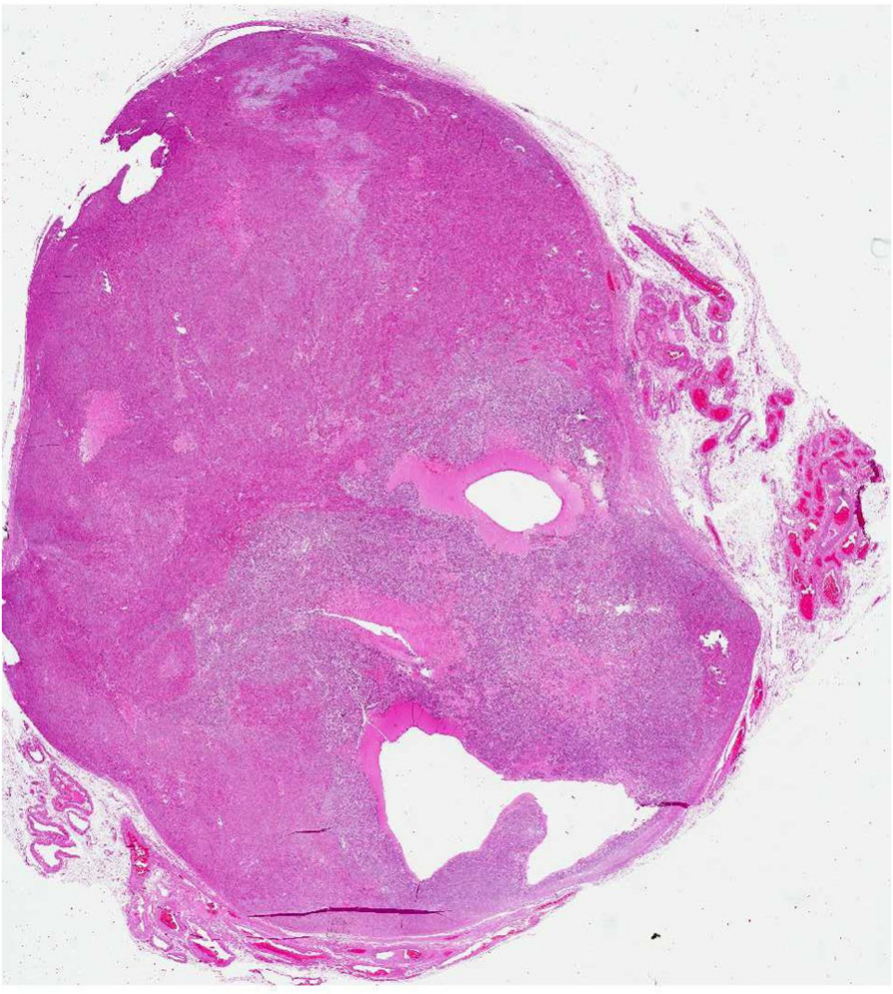
Whole mount histological section of the neoplasm with cystic-like areas containing mucoid-like material.

Macroscopically, the lesion, well circumscribed and firm, measures 5.1 cm by 4.0 cm and 3.4 cm in the three diameters. On the cut surface the lesion is whitish in color with mucoid-like areas (Figures 2 and 3). The lesion is separate from the testis and was attached to the spermatic cord.

**Figure 4:**
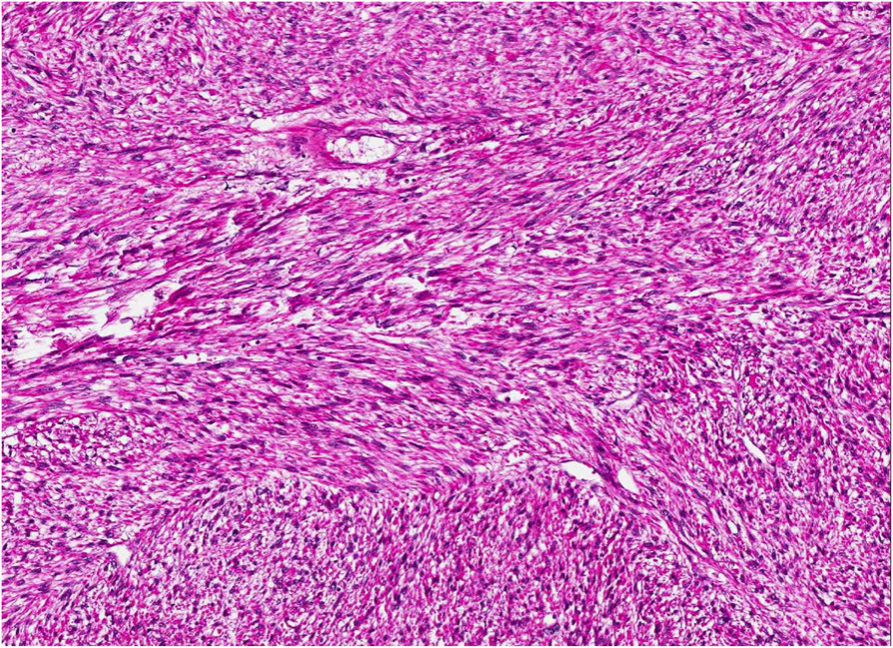
Spindle cell component of the neoplasm.

**Figure 5:**
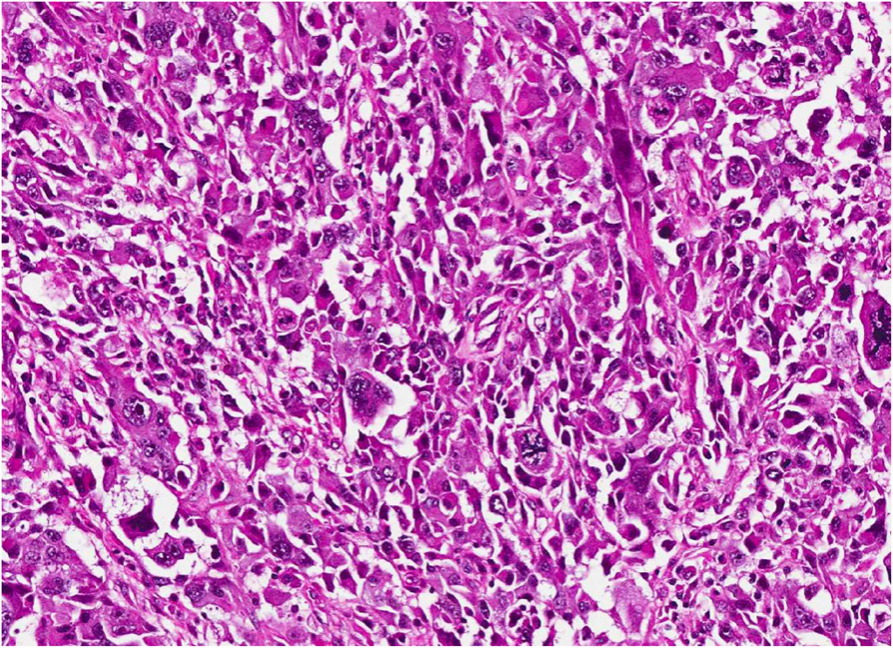
Pleomorphic area of the neoplasm.

**Figure 6:**
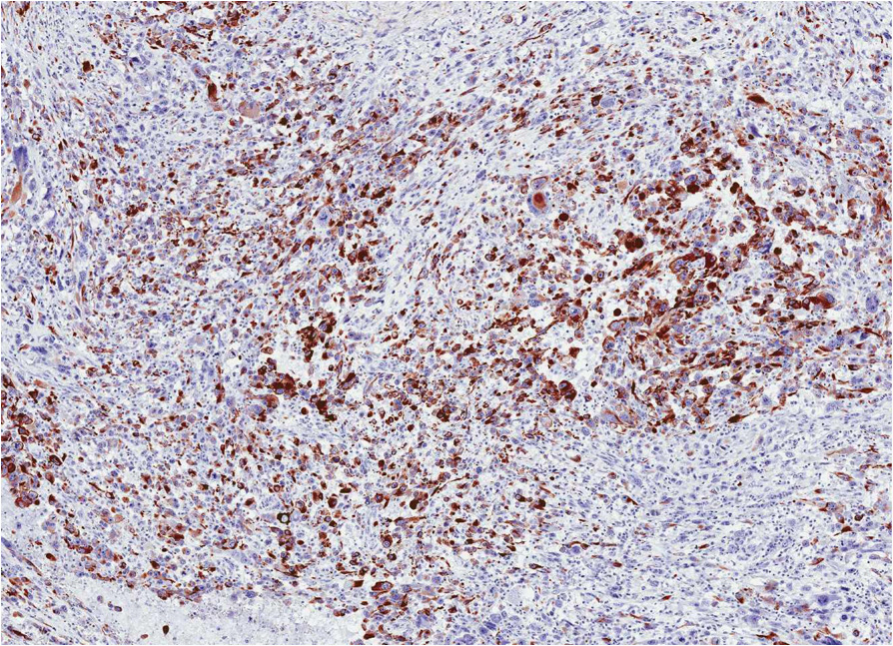
The neoplasm is intensely immunostained with an antibody to desmin.

Histologically, approximately 80% of the lesion is composed of spindled cells with often elongated, blunt-ended nuclei and variably eosinophilic cytoplasm (Figure 4). Areas with pleomorphic morphology are present (Approximately 20% of the lesion) (Figure 5). Necrosis is not present. However, cystic-like areas containing mucoid-like material are present. The level of mitotic activity is equal to 3/10 HPF in the areas with spindle cell morphology and to 12/10 HPF in the areas with pleomorphic morphology. The neoplasm stains strongly with antibodies to smooth muscle actin, muscle-specific actin, and desmin (Figure 6). CD34, cytokeratin, S-100 protein, MyoD1 and myogenin are negative. The final diagnosis was that a leiomyosarcoma of the spermatic cord, with grade 1 and grade 2 areas, stage pT2b cN0 and cM0. The patient has been followed up for 3 months with CT scans and shows no signs of recurrence.

## Discussion

Leiomyosarcoma accounts for 5%–10% of soft tissue sarcoma. Paratesticular LMS originates from the spermatic cord, the scrotum or the epididymis. The spermatic cord type arises from undifferentiated mesenchymal cells of the cremasteric muscle and vas deferens. The epididymal form originates from the smooth muscle surrounding the basement membrane of the epididymis. The scrotal type arises from the dartous layer. The first two aforementioned types drain into the retroperitoneal lymph nodes in contrast with the last type, which drains into the inguinal, external and internal iliac nodes. LMS is subdivided topographically into 3 groups: LMS of the deep soft tissue, LMS of the cutaneous and subcutaneous tissue and LMS of vascular origin. According to the AJCC Cancer Staging System, paratesticular LMS should belong to the deep subtype [14]. Its behavior is related to the site, histological grade of the lesion and the presence of nodal or distant metastases.

A recent review of 24 primary paratesticular LMS found 11 tumors in the testicular tunics, 10 in the spermatic cord, 1 in the scrotal subcutis, 1 in the dartos muscle, and 1 in the epididymis [6]. These were from men aged 34–86 years with a peak incidence in the sixth and seventh decade [15-20]. In the study by Coleman et al. [8], 22% of the patients presented with metastatic disease or subsequently developed distant sarcoma. A reported survival rate is 50%–80% [5].

Sonography should be the initial imaging modality since it can determine the origin of the lesion and even though the imaging characteristics are not adequate to reach a single diagnosis, the heterogeneous appearance along with the irregular, often increased vascularity of the tumor may allow the diagnosis of a sarcoma. Correlation with case history of the patients and CT/MR findings can further limit the differential diagnosis and lead to a better management of the patient. The majority of the LMSs are heterogeneous lesions, although some LMSs appear to be hypoechoic. Calcifications are not mentioned in the majority of the cases described. Colour Doppler ultrasonography shows either minimal, or increased vascularity. The appearance is mostly related to the size of the lesion and the differentiation of the mesenchymal components. A non-homogeneous mass with irregular, peripheral contrast enhancement and HU between 20 and 65, indicative of cystic, solid and calcified areas were found. A thickened and edematous spermatic cord with distended vessels was also depicted. The above CT findings parallel the sonographic ones. In addition to that, absence of areas with negative HU excluded the presence of fat within the lesion [21].

The above-mentioned features are pathologically correlated to necrotic areas within the tumor (cystic areas), infiltration of the epididymis (epididymis not visualized), origin from the spermatic cord (high position of the lesion within the scrotum), absence of testicular infiltration (definite testicular borders), neoplastic lesions within the spermatic cord (thick and edematous appearance) and infiltration of the vessels (vessel distention within the cord)

The morphologic range of LMSs is similar to that reported in other soft tissue sites, including a tumor with prominent myxoid areas [22], an inflammatory LMS [23], and a neoplasm with epithelioid foci [24,25]. The majority, however, display classic features of soft tissue LMS, i.e., perpendicularly oriented fascicles of cells with brightly eosinophilic cytoplasm containing delicate longitudinal fibrils and blunt-ended nuclei [26,27]. Although some lesions have numerous pleomorphic nuclei, typical features of smooth muscle differentiation are retained; even the pleomorphic nuclei usually remain blunt-ended and paired with brightly eosinophilic cytoplasm. Pleomorphic LMSs, however, are composed predominantly of areas resembling malignant fibrous histiocytoma (showing no differentiation) but with focal zones with typical features of smooth muscle differentiation on hematoxylin and eosin staining and the appropriate immunophenotype [28,29].

Immunohistochemistry is useful in confirming smooth muscle differentiation. Most cases stain strongly with antibodies to smooth muscle actin, muscle-specific actin, and desmin. Some also stain for CD34. Focal cytokeratin and S-100 protein positivity may occur, but myogenin stains have been negative. Spindle cell rhabdomyosarcomas in children can resemble LMSs. However, immunohistochemical stains for MyoD1 and myogenin are positive in the former and negative in the latter.

The NCI grading system identify three grades in LMS [30,31]. Grade 1 tumors lack necrosis, had <6 mitoses/10 HPF, and have only occasional pleomorphic nuclei. Grade 2 tumors have focal necrosis (<15%) and/or mitotic activity >6 mitotic figures/10 HPF or prominent nuclear pleomorphism. The key feature of grade 3 tumors is >15% necrosis, regardless of mitotic counts or numbers of pleomorphic nuclei. Various studies indicate the prognostic importance of grading LMSs in this site because whereas most examples are low grade and behave indolently, high-grade lesions are aggressive [6]. High-grade LMSs with pleomorphic nuclei can resemble dedifferentiated liposarcoma. This can be either identified by the presence of adjacent foci, which are sometimes very small, of well-differentiated liposarcoma, or excluded by finding more typical architectural or cytologic features of LMS with further sampling [6,2].

Simple excision is suboptimal as repeat wide excision has demonstrated microscopic residual disease in 27% of cases and therefore warrants an additional adjuvant treatment. Radical orchiectomy and funicolectomy (inguinal approach) is the cornerstone of treatment in the management of this neoplasm, but the reported survival rates indicate the need for additional treatment [5].

Comprehension of the pattern of spread is essential, but this task is difficult by the rare occurrence of this disease. The most common means of dissemination are by regional lymph nodes spread (external, common iliac, hypogastric and retroperitoneal lymph nodes), haematogenous metastases (most commonly to the lungs) and by local extension (local infiltration of the scrotum, inguinal canal or pelvis, along the pathway of vas deferens). Further series suggested that lymph node dissection (especially retroperitoneal) should not be performed unless enlarged lymph nodes are encountered on CT scans or palpated during surgery.

The literature concerning benefit of adjuvant therapy after radical surgery is inconclusive because of the small numbers. Recurrence after orchidectomy alone was common in earlier reports, and adjuvant radiation has been recommended to reduce locoregional failure [5].

Due to the high incidence of loco-regional recurrence in the lymph nodes, 2 different treatment alternatives, prophylactic retroperitoneal lymph node dissection (RPLND) and radiotherapy, have been tried. The proponents of RPLND indicate that there is a 29% risk of metastatic potential to regional lymph nodes. A review of 101 patients by Banowsky and Schultz described 29 cases of RPLND with lymph node involvement. Of these, 17 patients had isolated lymphatic dissemination. Despite such a high incidence of lymphatic spread, no report has yet shown a significant survival benefit from the addition of RPLND to radical orchidectomy [32]. A study from Massachusetts involved 18 patients who were subdivided into 2 groups of 9 patients. One group had surgery and the other group had surgery plus radiation therapy. Of the 9 patients treated with radical orchidectomy alone, 5 developed loco-regional failure, 2 of which were limited to lymph nodes. In contrast, there were no loco-regional recurrences among the 9 patients who received adjuvant radiation to the regional lymph nodes [9]. These findings, which are consistent with those of Catton and colleagues, suggest that adjuvant radiation may effectively control loco-regional microscopic disease [33].

In conclusion, spermatic cord leiomyosarcoma, although rare, should be included in the list of differential diagnoses for a firm-to-hard lump in the cord. Apart from radical orchi-funicolectomy, there has been added benefit of adjuvant radiotherapy to prevent any loco-regional lymph node recurrence.

## Competing interests

The authors declare that they have no competing interests.

## Authors' contribution

Drs ABG and LG: conception and design of the study. Dr RM: acquisition of data. Dr MS: drafting of the manuscript. Drs AL-B and LC: critical revision of the manuscript for important intellectual content. Dr RM: supervision. All authors read and approved the final manuscript.
